# Single- and Double-Loaded All-Suture Anchor Repairs of Anteroinferior Labral Tears Are Biomechanically Similar in a Cadaveric Shoulder Model

**DOI:** 10.1016/j.asmr.2022.07.011

**Published:** 2022-09-19

**Authors:** Byron Ellis, Todd Baldini, Elisabeth Geraghty, Eric McCarty

**Affiliations:** a University of Colorado-Denver, Aurora, Colorado, USA; b Department of Orthopedics, University of Colorado, School of Medicine, Aurora, Colorado, USA; c CU Sports Medicine and Performance Center, Boulder, Colorado, USA; d Department of Orthopedics, University of Colorado, School of Medicine, CU Sports Medicine and Performance Center, Boulder, Colorado, USA

## Abstract

**Purpose:**

To compare the biomechanical strength of single- versus double-loaded all-suture constructs in an anteroinferior glenoid labral repair.

**Methods:**

Anteroinferior labral lesions were created on 6 matched pairs of cadaveric shoulder specimens. Each shoulder in a matched pair was randomized to either receive capsulolabral repair with 3 single-loaded all-suture anchors or 3 double-loaded all-suture anchors. Immediately following capsulolabral repair, the specimens underwent mechanical testing, which included cyclic testing (5 N to 50 N for 500 cycles) and load-to-failure testing (rate of 15 mm/min). The gap formation between the repaired labrum and glenoid (measured at 1, 25, 100, and 500 cycles), the load at 2-mm gap formation, the maximum load at failure and the method of failure were recorded. Data were analyzed with paired Student *t* tests and Bonferroni correction factor.

**Results:**

The single and double all-suture constructs did not differ significantly in gap formation at any number of cycles, load to 2-mm gap formation (*P* = .75), or maximum load to failure (*P* = .46) between the 2 groups.

**Conclusions:**

In this study, single-loaded and double-loaded all-suture anchor constructs demonstrated comparable biomechanical performance and did not significantly differ in gap formation, load to 2-mm gap formation, or maximum load to failure when used in the capsulolabral repair of anteroinferior glenoid labral tears in human cadaveric specimens.

**Clinical Relevance:**

Although studies have evaluated the biomechanical properties of various arthroscopic labral stabilization techniques, the biomechanical properties of all-suture anchors with regard to labral stabilization are not well understood.

Anterior dislocation of the glenohumeral joint is a common entity in young and active individuals, especially those participating in contact sports and overhead activities.^1^ Younger individuals have a high propensity for recurrent anterior instability after an initial dislocation of the shoulder, especially with associated injuries to the capsulolabral structures of the shoulder. Bankart^2^ described the “essential lesion” that contributed to recurrent anterior instability but did not describe the lesion we know today as the “Bankart lesion.” Several biomechanical studies in the literature^3^, ^4^, ^5^, ^6^ determined that Bankart’s original “essential lesion” was not an isolated lesion but instead was a constellation of pathoanatomy of the capsuloligamentous structures of the shoulder that contributed to recurrent instability. Speer et al.^7^ demonstrated serially sectioning portions of the glenohumeral ligaments increased anterior translation in a cadaver model.

In 1981, Turkel et al.^8^ defined the inferior glenohumeral ligament as the primary static restraint to anterior translation. Several biomechanical studies have corroborated these findings since then,^9^, ^10^, ^11^, ^12^ which explains why current treatment concepts revolve around the necessity of capsulolabral stabilization to adequately treat recurrent anterior instability. Despite several technical advances over the last decade mirroring the rise of arthroscopically guided stabilization of the glenoid labrum, there is no consensus in the literature on a specific surgical technique to address this entity using an arthroscopic approach.^13^ Although studies have evaluated the biomechanical properties of various arthroscopic labral stabilization techniques, few have studied the biomechanical properties of all-suture anchors with regards to labral stabilization.

Recently, all-suture anchors have been developed for use in shoulder-stabilization surgery. In addition to providing adequate fixation and reliable clinical results, all-suture anchors use smaller pilot holes, which provide the advantages of decreased removal of bone and decreased glenoid volume compared to traditional tap or screw-in suture anchors.^14^^,^^15^ Manufacturers have started producing all-suture anchors with an additional suture (double-loaded). Theoretically, each suture can capture a larger section of the capsulolabral complex through a single anchor point in the glenoid. In addition, in the event of suture anchor pullout, the absence of a hard loose body that can cause chondral injury in the joint is appealing. Double-loaded suture anchor applications in other aspects of shoulder surgery such as bicep tenodeses and SLAP and rotator cuff repairs have shown promise in regard to improved fixation strength compared with single-loaded constructs in the cadaveric model.^16^, ^17^, ^18^ Logically, increasing the amount of suture material across the repair would increase the initial fixation strength of arthroscopic stabilization of a glenoid capsulolabral lesion.

The purpose of this study is to compare the biomechanical strength of single- versus double-loaded all-suture constructs in an anteroinferior glenoid labral repair. The null hypothesis was that there would no significant biomechanical advantage of double-loaded all-suture anchors compared with single-loaded all-suture anchors.

## Methods

Six matched pairs of cadaver shoulders including the scapula with all soft tissue, the humeral head, and clavicle were obtained from a tissue bank (Science Care, Phoenix, AZ). There were 3 male donors (ages 60, 61, and 62 years) and 3 female donors (ages 45, 55, and 61 years). The cadaver shoulders were purchased through a grant from the University of Colorado, Department of Orthopedics' Resident Research Committee. The study satisfied the institutional review board’s requirements for cadaver research.

The specimens were thawed to room temperature before dissection and mechanical testing. All soft tissue except the glenoid labrum and joint capsule was carefully removed from the scapula. The anteroinferior glenoid labrum was marked at the 3- and 6-o’clock positions on the right glenoid (9- and 6-o’clock on the left glenoid). An elevator was used to gently lift the edge of the labrum off the glenoid whereas a #15 scalpel was used to cut the labrum from the glenoid from 3- to 6-o’clock on the right scapula (9- to 6-o’clock on the left). Since the 2 anchor constructs were being compared with a paired *t* test, the left and right shoulders from a singular donor required identical lesions. Assuming the glenoids of matched left and right shoulders were the same size, creating tears based on clock face allowed for the creation of identical defects on matched pairs. Because glenoid size could differ between donors, the exact labral lesion length varied between donors but was consistently proportioned in all specimens from 3 to 6 o’clock on right shoulders and 9 to 6 o’clock on left shoulders. The capsule and remaining labrum outside the 3- to 6-o’clock or 9- to 6-o’clock position was removed to ensure strength testing of just the isolated repair, and not the strength of the repair and labrum.

**Fig 1:**
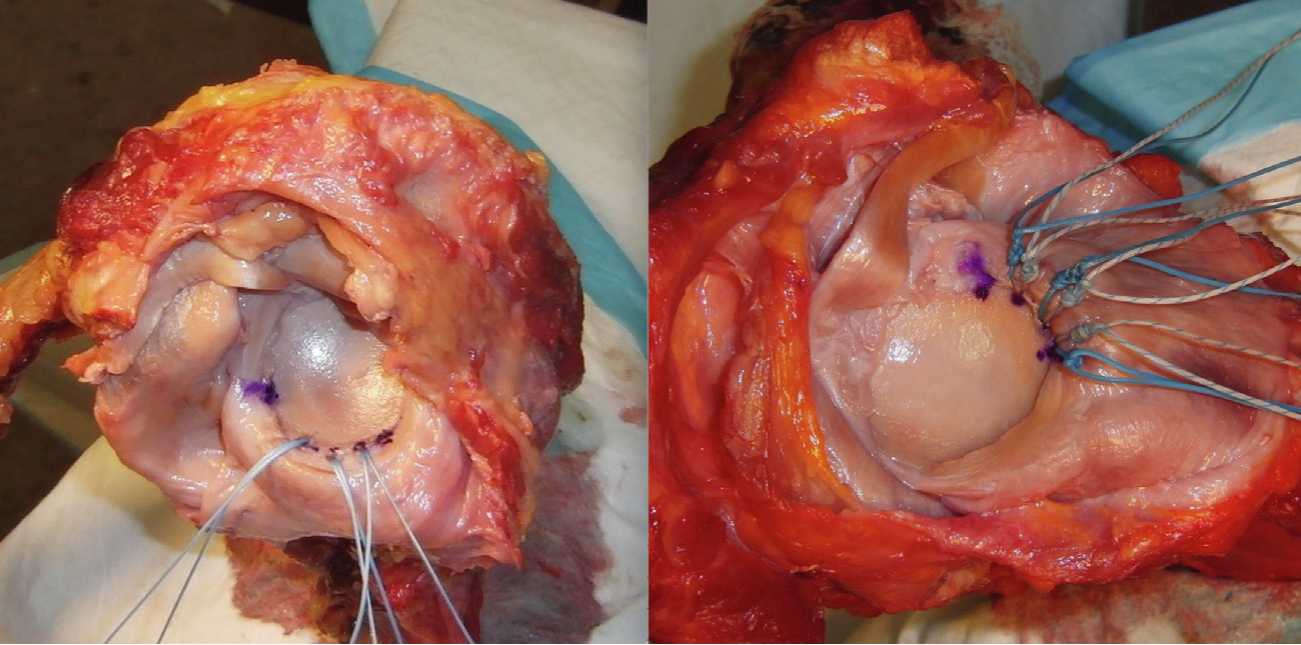
Single-loaded all-suture anchor labral repair (left) compared with the double-loaded all-suture anchor labral repair (right).

The simulated labral tears were repaired with three 2.9-mm double-loaded all-suture anchors (JuggerKnot; Zimmer-Biomet, Warsaw, IN). The anchors were loaded with #2 MaxBraid suture. The first anchor was placed at the 5:30-o’clock position on the right scapula (6:30 o’clock on the left scapula) with the appropriately sized drill bit and guide per the manufacturer’s instructions. The drill guide was placed onto the glenoid and a 3-mm diameter, 20-mm deep pilot hole was drilled. The JuggerKnot all-suture anchor was then secured into the pilot hole. The next 2 anchors were each placed 4 mm edge to edge from the previous anchor. The initial anchor placed in the 5:30-o’clock position is the inferior anchor. The anchor closest to the 3-o’clock position is the superior anchor and the anchor between the 2 anchors is called the middle anchor. The specimens in each matched pair were assigned randomly to either be a single-loaded or a double-loaded repair. For the single-loaded repair group, after the anchor was seated, one strand of suture was carefully removed from the anchor. Although single-loaded all-suture anchors come in a smaller 1.7-mm size, we chose to remove a suture from the double-loaded anchor to keep the anchor size and the amount of glenoid bone volume occupied consistent between groups. The sutures were passed through the labrum with a Mayo half taper needle and were tied with a Duncan sliding knot and 3 alternating half hitches (Fig 1).

**Fig 2:**
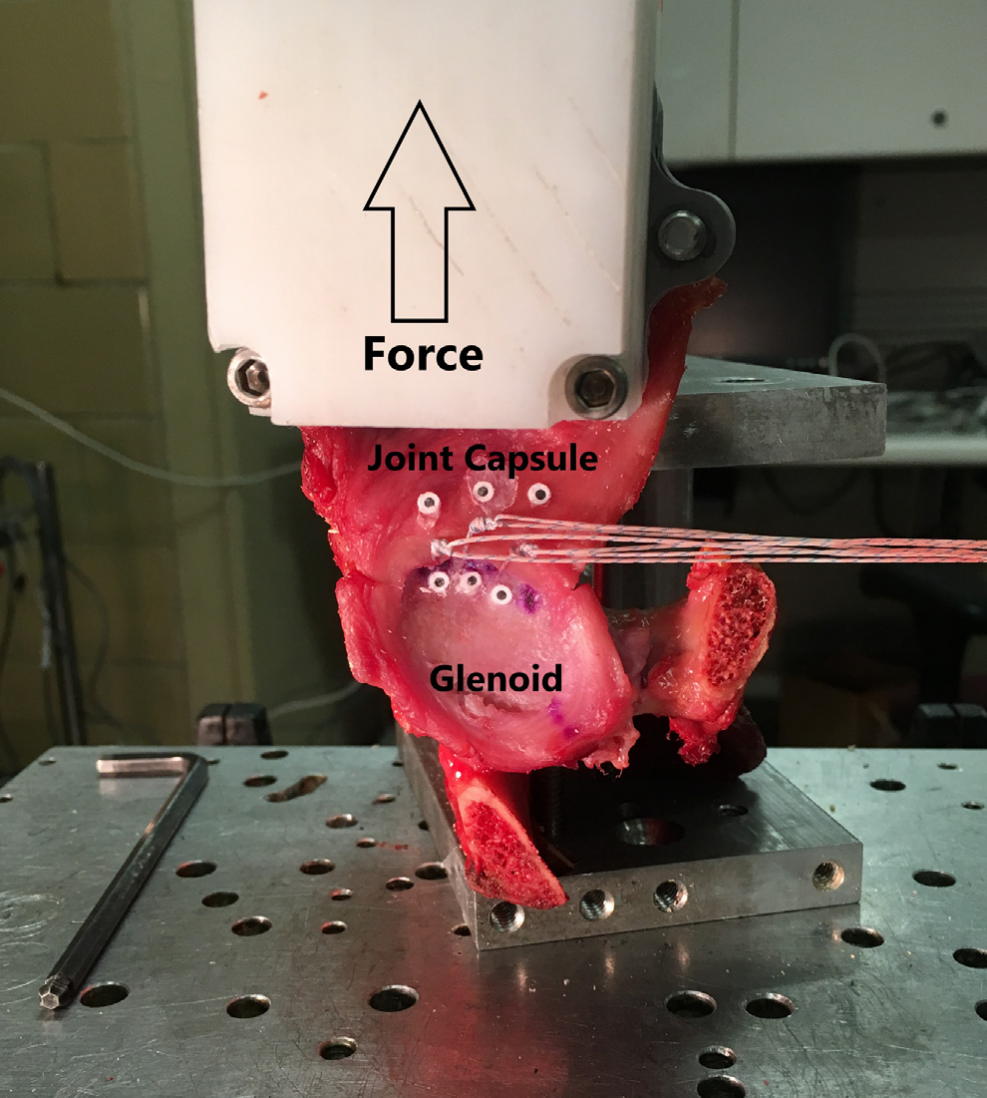
Test setup showing the specimen mounted on the base of the Instron, the optical markers, and the cryo-clamp. (The sutures are being held to the side to prevent them from blocking the cameras view of the optical markers.)

For mechanical testing, the glenoid was mounted on the base of an Instron servohydraulic test machine (Instron Corp., Norwood, MA) as described by Nho et al.^19^ and Judson et al.^20^ A cryo-clamp was attached to the joint capsule 15 mm superior to where the sutures passed through the joint capsule to affix the anteroinferior portion of the glenoid in the superior position (Fig 2). Optical markers were placed on the glenoid 1 mm inferior to and 1 mm superior to the anchors on the joint capsule. The cryo-clamp was then attached to the actuator of the Instron (Fig 2). This setup generated a force in the anteroinferior direction to induce maximum stress on the repair to simulate a dislocation and repair failure.

The following biomechanical protocol has been used and published in previous studies by our group and other laboratories.^17^^,^^19^, ^20^, ^21^, ^22^ The labrum was first preloaded to 5 N for 2 minutes, and then loaded from 5 N to 50 N with a sine wave at 0.25 Hz for 500 cycles. Cyclic loading is clinically relevant because the sutures should not exhibit creep from the daily motion of a shoulder. After cyclic testing, the labrum was loaded to failure at a rate of 15 mm/minute and the method of failure was noted. Maximum load to failure represents a violent force resulting in a dislocation. During testing, the optical markers were recorded using a Sentech model STC-MBCM401U3V camera (Sentech America, Inc., Carrollton, TX) with MaxTRAQ 2D motion analysis software at 25 frames per second (Innovision Systems, Inc., Columbiaville, MI). The actuator load and displacement were recorded at 25 Hz. The gap formation between the optical markers at each anchor was measured at 1, 25, 100, and 500 cycles with the MaxTRAQ 2D software. During load to failure testing, the load at the 2-mm gap formation, thought to be an indicator of clinical failure, and the ultimate load to failure were reported.^19^

### Statistical Analysis

A power analysis was conducted to determine the number of specimens required to detect a significant difference with α = 0.05 and 1 – β = 0.80 using the technique described by Dell et al.^23^ To detect an 80-N difference in ultimate load to failure with a standard deviation of 60N, a sample size of n = 6 specimens per group was calculated.^23^ The gap formations at the 3 anchor positions (superior, middle, and inferior) were analyzed with a paired Student *t*-test and Bonferroni correction factor at 1, 25, 100, and 500 cycles with a significance level set at *P* < .0125 (0.05/4). A paired Student *t*-test was used to analyze the clinical and ultimate load to failure data with the significance level set at *P* < .05.

## Results

**Fig 3:**
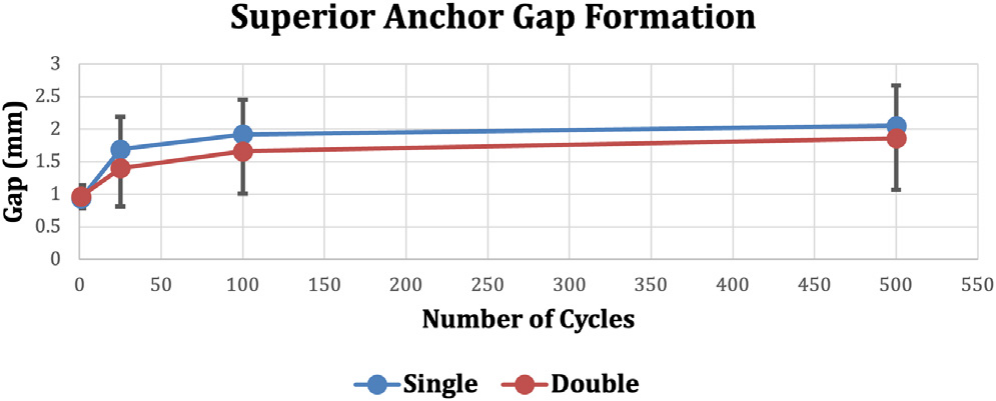
Superior anchor gap formation during cyclic loading. First cycle, *P* = .36; 25th cycle, *P* = .39; 100th cycle, *P* = .46; 500th cycle, *P* = .66.

**Fig 4:**
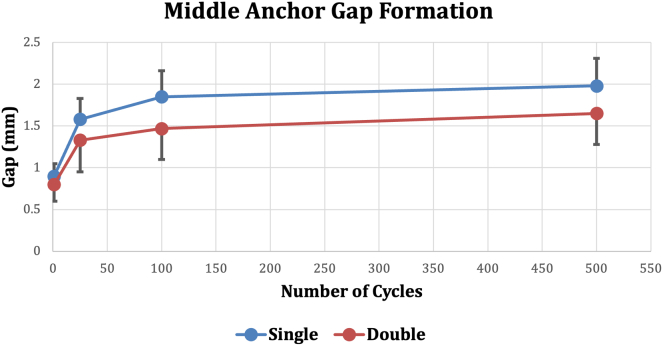
Middle anchor gap formation during cyclic loading. First cycle, *P* = .24; 25th cycle, *P* = .16; 100th cycle, *P* = .05.; 500th cycle, *P* = .05

**Fig 5:**
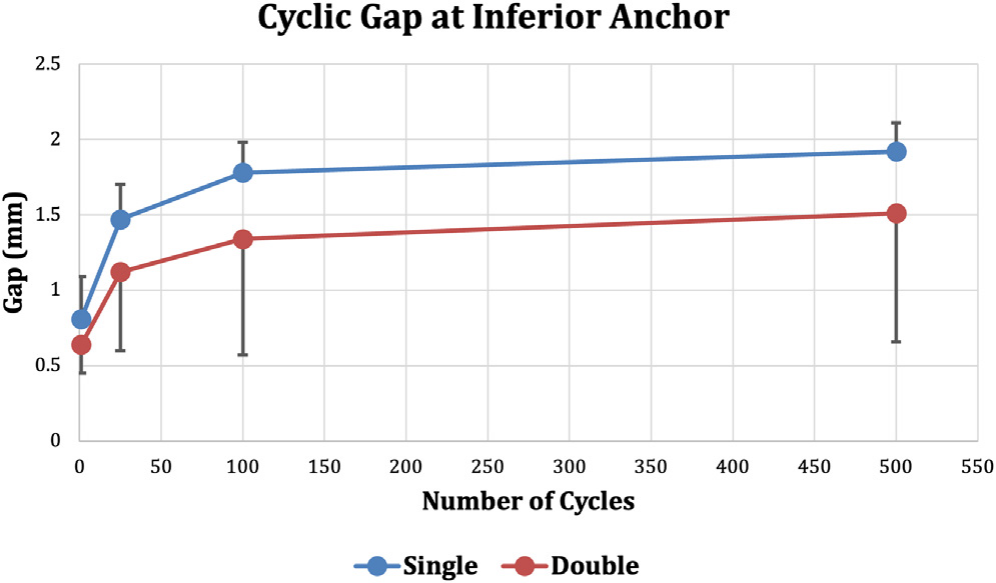
Inferior anchor gap formation during cyclic loading. First cycle, *P* = .34; 25th cycle, *P* = .23; 100th cycle, *P* = .28; 500th cycle, *P* = .30

There were no significant differences in the gap formation for either anchor construct at any number of cycles (*P* values shown in Fig 3, Fig 4, Fig 5). All data shown are mean ± 1 standard deviation. Load to 2-mm gap formation in the single-loaded anchor group (101.8 ± 31.7 N) and the double-loaded anchor group (97.2 ± 21.0 N) were not significantly different (*P* = .75). Maximum load to failure in the single-loaded group (264.2 ± 64.1 N) and the double-loaded group (234.2 ± 48.7 N) were not significantly different (*P* = .46). Two of the double-loaded suture anchors and one of the single-loaded suture anchors failed by the anchor pulling out of the bone. All other specimens failed by the suture pulling through the labrum (n = 9). All failures occurred at loads greater than the load to 2-mm gap formation; 259% greater for the single-loaded suture anchor and 241% greater for the double-loaded suture anchor.

## Discussion

The findings demonstrated that 3 single-loaded and 3 double-loaded all-suture anchor constructs did not significantly differ in gap formation, load to clinical failure gap formation, or maximum load at failure when used in the repair of anteroinferior glenoid labral tears in human cadaveric specimens. This suggests that increasing the amount of suture material across the glenoid labral repair, by using double-loaded versus single-loaded all-suture anchors, does not significantly improve fixation strength or provide additional benefit in anteroinferior labral repair.

Techniques and implants have expanded and evolved to address glenoid labral tears, which has resulted in improved outcomes. Current recommendations include the use of a minimum of 3 conventional single-loaded anchors in the anteroinferior glenoid to maximize capsular fixation.^24^^,^^25^ Given some of the advantages of all-suture anchors compared with other designs, orthopedic surgeons have a vested interest in the development of an all-suture anchor technique for use in glenoid labrum repair. As all-suture anchors gain popularity in capsulolabral repairs, it is important to establish anchor placement concepts for these types of anchors such as anchor number and single versus double-loaded fixation.

Biomechanical comparisons of varying anchor constructs have been published extensively in the orthopedic literature. However, the majority of these have focused on anchor, suture, and knot properties, without comparisons between different suture constructs (i.e., single vs double-loaded) using identical anchors and technique.^20^^,^^24^^,^^26^, ^27^, ^28^, ^29^, ^30^ Our biomechanical study compared single-loaded and double-loaded all-suture anchor labral repairs in a paired fashion, which allowed for direct head-to head comparison of one type of implant. Our study used paired specimens to normalize for variability typically seen in capsular tissue strength and glenoid bone stock seen in unpaired specimens. All repairs were performed using the same suture configuration, instruments, suture material, and arthroscopic knot-tying technique by the same orthopedic surgeon. Lastly, optical tracking using the MaxTRAQ 2D software allowed for more accurate displacement measurements.

A similar biomechanical study by Mazzocca et al.^26^ compared the use of an all-suture anchor versus a classic solid anchor in glenoid labral repair. Their study found that the SutureTak 3.0-mm classic solid anchor required a significantly greater ultimate load at 2 mm of labral displacement compared with the all-soft suture anchor, 84.1 ± 19.0 N and 39.2 ± 10.6 N, respectively.^26^ In our study, the load to 2-mm gap formation for the single (101.8 ± 31.7 N) and double-loaded (97.2 ± 21.0 N) suture anchors were comparable with the classic solid anchors in the study by Mazzocca et al.^26^ The cadaver specimens in this study had similar demographic characteristics (mean age: 61 ± 9.4 years, 2/6 male) as the present study and therefore differences in ultimate load to 2-mm gap formation are likely due to other factors. The study by Mazzocca et al.^26^ used a JuggerKnot 1.4 mm made up of a stiff section of suture constructed of a short strand of polypropylene, and a single size No. 1 ultrahigh-molecular-weight polyethylene suture inserted into this anchoring suture. In contrast, our study used a JuggerKnot 2.9-mm double-loaded all-suture anchor loaded with a #2 MaxBraid suture. The all-suture anchor in our study is larger and the suture within the anchor is stouter, which could contribute to the greater amount of force needed to displace the labral repair 2 mm. Of note, the study by Mazzocca et al.^26^ only used 2 suture anchors, whereas the current study used 3 suture anchors for labral repair testing, which also may account for these differences.^26^^,^^27^

A study by Nho et al.^19^ analyzed single-loaded versus double-loaded suture anchors in multiple suture configurations in a cadaveric model. They demonstrated that ultimate load to failure in single- (184 ± 64.5 N) and double-loaded (216.7 ± 61.7 N) anchor constructs was not significantly different. The findings of the current study agree with Nho et al.’s conclusions but measured slightly greater maximum loads to failure in both groups (single 264.2 ± 64.1 N, double 234.2 ± 48.7 N, *P* = .46). Unlike our study, the protocol in Nho et al.^19^ did not use matched pairs, which may have introduced differences in glenoid bone stock and capsular tissue quality. Although the cadaver specimens had a greater proportion of specimens from male donors (5/5), their specimens had a slightly greater mean age (72.8 ± 13 years), which could explain the slightly lower maximum loads to failure. In addition, they had a smaller sample size (n = 10 instead of n = 12) and used fewer anchors for the repair (2 instead of 3 anchors) compared with our study. These factors may contribute to the slight difference in our maximum load to failure values.

A study by Barber et al.^28^ tested JuggerKnot 1.4-mm single-loaded anchors using porcine femur and demonstrated the all-suture construct had a median force to failure of 239.1 N with a range of 215 to 263 N. Despite only using 2 anchors and a smaller suture size, this study had similar maximum load to failure values as the current study. Porcine femur specimens differ from intrinsic human bone characteristics and is likely the explanation for this observed difference.

A study by Kamath et al.^31^ demonstrated that a Bankart repair with 2 double-loaded suture anchors offers a significantly greater ultimate load to failure (944 ± 231 N) compared with a repair with 3 single-loaded anchors (784 ± 287 N). Despite similar cadaver demographics (mean age: 53.6 ± 7.1 years), this study measured a greater load to failure compared with the current study, which may be due to the type of anchor used. Kamath et al^31^ used solid suture anchors, which have historically high loads to failure given their stiff grounding (i.e., screw)^29^ compared with all-suture anchors. In addition, the study by Kamath et al^31^ left the rest of the labrum intact after creating the labral lesion. In contrast, the current study removed the rest of the labrum after creating the labral lesion which may explain the lower failure loads.

Despite small variations in ultimate load to 2-mm gap formation and failure, the majority of studies demonstrated similar modes of failure. In all of the shoulders in the current study, the failure occurred with maximum load through the bone or soft tissue of the cadaver (3/12 anchor pullout and 9/12 suture pulling through the joint capsule). Similarly, in the study from Mazzocca et al.^26^, 22 of 24 failures resulted from anchor pullout and 2 of 24 resulted from suture pull-through. In the study by Nho et al.,^19^ 8 of 10 failures occurred due to anchor pullout, 1 of 10 due to glenohumeral junction tear, and 1 of 10 due to capsule rupture. In the study from Kamath et al.,^31^ 6 of 18 failures resulted from anchor pullout, 7 of 18 from capsule/labrum interface rupture, 3 of 18 from scapula/glenoid fracture, and only 2 of 18 from labral tear. The study by Barber et al.^28^ was the only one that noted the mode of failure to be mostly suture breaking (11/20) followed by anchor pullout (9/20). However, the study by Barber et al.^28^ created a maximum load on the anchor by pulling on the suture instead of pulling on the joint capsule which could explain these differences. Almost all modes of failure in these studies occurred with maximum load through bone or soft tissue, which would suggest that the anchors and sutures themselves were likely not failing. Instead, the results from the present study and previous literature established that the intrinsic properties of the sutures, knots, and anchors are all stronger than the tissues, in both single- and double-loaded constructs. As a result, differences in ultimate load values between studies might instead reflect variations in biomechanical testing methods^32^ or intrinsic qualities of cadaver tissue.

### Limitations

Due to a small sample size, there is a possibility of a type 2 error. In addition, bone density tests were not performed to prove the presumed osteopenic state of the cadaveric specimens. There were several limitations inherent to a biomechanical study design that limited comparison to in vivo labral repair. For example, dissection of the cadavers removed possible natural static capsular restraints such as the negative intraarticular hydrostatic pressure supplied by the intact capsule and viscous characteristics of synovial fluid. Dissection also removed the natural dynamic restraints of the rotator cuff musculature which may add to the overall compression and stability of an in vivo repair of the glenoid labrum. Furthermore, the Instron servohydraulic apparatus only stressed the simulated labral repairs by pulling at a 90° angle to the glenoid face, which does not represent the multiple directions of force vectors seen by an intact glenohumeral joint. Finally, as a biomechanical study, this study does not replicate the natural healing process that is involved over several weeks to months postoperatively after a capsulolabral reconstruction. Testing strength at time zero in this study does not necessarily reflect the added soft-tissue healing strength that develops over time in addition to the suture fixation strength.

## Conclusions

In this study, single-loaded and double-loaded all-suture anchor constructs demonstrated comparable biomechanical performance and did not significantly differ in gap formation, load to 2-mm gap formation, or maximum load to failure when used in the capsulolabral repair of anteroinferior glenoid labral tears in human cadaveric specimens.

## Supplementary Data

ICMJE author disclosure forms
